# Comparison of Carboplatin With Cisplatin in Small Cell Lung Cancer in US Veterans

**DOI:** 10.1001/jamanetworkopen.2022.37699

**Published:** 2022-10-20

**Authors:** Ibrahim Azar, Omid Yazdanpanah, Hyejeong Jang, Adam Austin, Seongho Kim, Jie Chi, Samer Alkassis, Biplab K. Saha, Amit Chopra, Kristoffer Neu, Syed Mehdi, Hirva Mamdani

**Affiliations:** 1 Barbara Ann Karmanos Cancer Institute, Department of Oncology, Wayne State University School of Medicine, Detroit, Michigan; 2 IHA Hematology Oncology, Pontiac, Michigan; 3 Division of Pulmonary and Critical Care Medicine, University of Florida, Gainesville; 4 Division of Pulmonary and Critical Care Medicine, Albany Medical Center, Albany, New York; 5 Division of Pulmonary and Critical Care Medicine, Albany Veteran Affairs Medical Center, Albany, New York; 6 Division of Medical Oncology, Albany Veteran Affairs Medical Center, Albany, New York

## Abstract

**Importance:**

The current standard of care for the treatment of small cell lung cancer (SCLC) is concurrent chemoradiation for patients with limited-stage SCLC (LS-SCLC) and chemoimmunotherapy for extensive-stage SCLC (ES-SCLC). The backbone of chemotherapy regimens in both is a platinum-etoposide doublet: cisplatin is traditionally the preferred platinum agent in the curative intent setting, whereas carboplatin is preferred in ES-SCLC because of its favorable toxicity profile.

**Objective:**

To determine whether cisplatin is associated with better survival outcomes than carboplatin in treating LS-SCLC and ES-SCLC.

**Design, Setting, and Participants:**

In this cohort study, data were compiled from the National Veterans Affairs Central Cancer Registry for patients with SCLC who received platinum-based multiagent chemotherapy between 2000 and 2020 for ES-SCLC and 2000 and 2021 for LS-SCLC. Only patients with pathologically confirmed cases of LS-SCLC who received concurrent chemoradiation and ES-SCLC who received chemotherapy were included.

**Main Outcomes and Measures:**

The primary end point was overall survival (OS). The secondary end points included OS by Eastern Cooperative Oncology Group performance status, age, and laterality. Interval-censored Weibull and Cox proportional hazard regression models were used to estimate median OS and hazard ratios (HRs), respectively. Survival curves were compared by a Wald test.

**Results:**

A total of 4408 SCLC cases were studied. Most patients were White (3589 patients [81.4%]), male (4252 [96.5%]), and non-Hispanic (4142 [94.0%]); 2262 patients (51.3%) were 60 to 69 years old, followed by 1476 patients (33.5%) aged 70 years or older, 631 patients (14.3%) aged 50 to 59 years, and 39 patients (0.9%) aged 30 to 49 years. Among 2652 patients with ES-SCLC, 2032 were treated with carboplatin-based therapy and 660 received cisplatin; the median OS was 8.45 months (95% CI, 7.75-9.20 months) for cisplatin and 8.51 months (95% CI, 8.07-8.97 months) for carboplatin (HR, 1.01; 95% CI, 0.91-1.12; *P* = .90). Subset analysis showed no survival difference between the 2 agents in different age or performance status groups except for patients aged 70 years and older, for whom the median OS was 6.36 months (95% CI, 5.31-7.56 months) for cisplatin and 8.47 months (95% CI, 7.79-9.19 months) for carboplatin (HR, 0.77; 95% CI, 0.61-0.96; *P* = .02). Multivariable analysis of performance status and age did not show a significant difference in survival between the 2 groups (HR, 0.96; 95% CI, 0.83-1.10; *P* = .54). Of 1756 patients with LS-SCLC, 801 received carboplatin, and 1018 received cisplatin. The median OS was 26.92 months (95% CI, 25.03-28.81 months) for cisplatin and 25.58 months (95% CI, 23.64-27.72 months) for carboplatin (HR, 1.04; 95% CI, 0.94-1.16; *P* = .46). The median OS was not significantly different between 2 agents according to cancer stage (I-III), performance status, and age groups. A multivariable analysis of factors associated with OS accounting for stage (I-III), performance status, and age did not demonstrate a significant difference in survival between carboplatin and cisplatin in patients with LS-SCLC (HR, 0.995; 95% CI, 0.86-1.15; *P* = .95).

**Conclusions and Relevance:**

Cisplatin is not associated with a survival advantage over carboplatin among patients with either ES-SCLC or LS-SCLC, irrespective of performance status and age. The favorable toxicity profile of carboplatin and comparable OS support its use in both LS-SCLC and ES-SCLC in clinical practice and may allow more room for combination with novel treatment strategies in clinical trials.

## Key Points

**Question:**

What is the platinum agent of choice in treating small cell lung cancer (SCLC), cisplatin or carboplatin?

**Findings:**

In this cohort study of 4408 patients at US Veteran Affairs hospitals, there was no significant difference in overall survival (OS) between those treated with carboplatin or cisplatin. For 2652 patients with extensive-stage SCLC, the median OS was 8.45 months for cisplatin and 8.51 months for carboplatin, and for 1756 patients with limited-stage SCLC, the median OS was 26.92 months for cisplatin and 25.58 months for carboplatin.

**Meaning:**

These findings suggest that cisplatin is not associated with a survival advantage over carboplatin in either extensive-stage or limited-stage SCLC, but carboplatin use should be favored because of its favorable toxicity profile.

## Teaser

This cohort study examines whether cisplatin is associated with better outcomes than carboplatin in limited-stage and extensive-stage small cell lung cancer.

## Introduction

Small cell lung cancer (SCLC) is a distinct subtype of lung cancer with poorly differentiated neuroendocrine features characterized by rapid doubling time and early development of widespread metastases.^1^ SCLC constitutes nearly 14% of all pulmonary malignant cancers and almost exclusively involves cigarette smokers. The incidence of SCLC is declining in the US, reflecting the decreasing use of tobacco.^2^ However, there has been no improvement in survival since the 1980s.^3^ SCLC has been classified into limited and extensive stages since publication of the results of the Veterans Administration (VA) Lung Study Group in 1957.^4^ Limited-stage SCLC (LS-SCLC) is tumor confined to the ipsilateral hemithorax and regional nodes that can be included in a single tolerable radiotherapy port. Extensive-stage SCLC (ES-SCLC), on the other hand, extends beyond a single hemithorax, including malignant pleural or pericardial effusions and hematogenous metastases. At the time of diagnosis, 70% of patients have extensive-stage disease.^2,5^ More recently, the International Association for the Study of Lung Cancer has recommended the adoption of the American Joint Committee on Cancer staging system based on more accurate prognostic accuracy.^6^ However, management has largely been based on the VA Lung Study Group staging.

Although immunotherapy and targeted agents have revolutionized the treatment of non–small cell lung cancer and greatly improved survival, progress in SCLC has, unfortunately, been more modest.^3,7,8^ Traditionally, concurrent chemoradiation is recommended for the management of LS-SCLC, and chemoimmunotherapy is recommended for ES-SCLC. The backbone of chemotherapy regimen in both LS-SCLC and ES-SCLC is platinum-etoposide doublet.^9,10^ In the 1990s, intensification trials^11,12^ failed to show any benefit over a platinum double in ES-SCLC. More recently, the IMpower133^13^ and the CASPIAN^14^ trials tested the addition of checkpoint inhibitors, atezolizumab and durvalumab, to chemotherapy with improvement in median overall survival (OS) of 2 and 3 months, respectively. However, whether cisplatin and carboplatin are equally effective in the treatment of SCLC is still controversial. In 2012, the COCIS meta-analysis^15^ found no survival differences between carboplatin-based and cisplatin-based regimens in ES-SCLC. Therefore, carboplatin is frequently preferred over cisplatin in the ES-SCLC setting, owing to its favorable toxicity profile with decreased renal, neurologic, otologic, and emetogenic toxic effects. However, cisplatin use remains frequent in fit and young patients with ES-SCLC. Among CASPIAN trial patients,^14^ 25% received cisplatin, whereas the IMpower133 study^13^ restricted chemotherapy to carboplatin only. As patients with LS-SCLC are treated with curative intent, National Comprehensive Cancer Network (NCCN) guidelines and most expert opinion recommend cisplatin as the preferred platinum agent. Unlike ES-SCLC, there are no phase 3 clinical trials comparing carboplatin and cisplatin in LS-SCLC. The majority of data in this space are derived from small phase 2 trials.^16,17^ In this retrospective cohort study, we attempt to evaluate the efficacy of carboplatin vs cisplatin in both LS-SCLC and ES-SCLC by querying the large National VA Cancer Cube Registry with the aim of comparing OS of patients treated with cisplatin vs carboplatin.

## Methods

This cohort study was reviewed and approved by the institutional review board committee at the Albany Stratton VA Medical Center. The study followed the Strengthening the Reporting of Observational Studies in Epidemiology (STROBE) reporting guideline. Consent was not obtained because the data were anonymous, in accordance with 45 CFR §46. Nationwide data from the National VA Cancer Care Cube Registry (CCCR)^18^ were analyzed. The main data source for the CCCR is the Oncology Domain tables on the Corporate Data Warehouse raw server, which is updated every 2 weeks. The Oncology Domain tables are created from the VISTA OncoTrax software package. The registry was accessed on April 14, 2020, for ES-SCLC and January 23, 2021, for LS-SCLC, and data input after this date are not included in this study. Unique cases of SCLC through all accession years were analyzed. The near totality of cases were entered after January 1, 2000. The registry defines unique cases as those with the same combination of the following data points: patient Social Security number, diagnosis date, primary tumor site, sequence number, *International Classification of Diseases for Oncology, Third Revision* histology code, grade differentiation identification, and laterality. The registrar further classifies cases according to abstract status. Complete abstract status indicates that all data points have been entered by the tumor registrar for that case. Only cases with complete abstract status were considered for this study, and cases with incomplete data were excluded. The cancer primary site (lung, small cell) was identified by a computed VISTA field that recorded the primary site or group major body system. A data set of SCLC was generated by application of the above qualifiers: unique cases, complete abstract, cancer primary site (lung), *International Classification of Diseases for Oncology, Third Revision* histology code consistent with SCLC, all accession years, and cancer stage (ie, stage I-III for LS-SCLC and stage IV for ES-SCLC).

Demographic data on the CCCR, including age at diagnosis, sex, and survival, were generated from the VA Health Eligibility Center demographic file. Survival in the CCCR was defined as less than 1 year, 1 to 5 years, 5 to 10 years, 10 to 15 years, and greater than 15 years. Race and ethnicity were derived from the Corporate Data Warehouse on the basis of information provided by patients at initial contact with the VA hospital and are reported in this study to provide a complete demographic profile of the cohort. Only cases where the first course of treatment was chemoradiation in LS-SCLC and chemotherapy in ES-SCLC were included. Chemotherapeutic agents administered as part of the first course of treatment were analyzed. Up to 5 chemotherapy agents can be recorded in VISTA OncoTrax as part of a cancer case’s first course of treatment. Only patients who received either carboplatin or cisplatin as part of multiagent chemotherapy in stage IV and multiagent chemoradiotherapy in stages I to III were considered. The *Surveillance, Epidemiology and End Results Self-Instructional Manual for Tumor Registrars, Book 8, Antineoplastic Drugs, Third Edition* is the data source for the chemotherapy agents.

### Statistical Analysis

Patient characteristics were summarized with count and percentage and compared between groups by Fisher exact tests. A parametric Weibull proportional hazards regression analysis for interval-censored data was performed to estimate OS rate, median OS, and their associated 95% CIs for each group. An interval-censored Cox proportional hazard model was further used to estimate hazard ratios (HRs) using the iterated conditional model algorithm with a constrained gradient ascent step. The multivariable analysis was also performed by an interval-censored Cox proportional hazard model. The SEs and 95% CIs of HRs were calculated using 1000 bootstrap replicates, and a Wald test was used to compare between survival curves. All *P* values are 2-sided with a significance level of *P *< .05. Data were analyzed using R statistical software version 4.1.2 (R Project for Statistical Computing).

## Results

A total of 4408 SCLC cases were studied. Most patients were White (3589 patients [81.4%]), male (4252 [96.5%]), and non-Hispanic (4142 [94.0%]). Overall 2262 patients (51.3%) were 60 to 69 years old, followed by 1476 patients (33.5%) aged 70 years or older, 631 patients (14.3%) aged 50 to 59 years, and 39 patients (0.9%) aged 30 to 49 years.

### Patients With ES-SCLC

A total of 2652 patients with ES-SCLC were included in this study; 2032 patients were treated with carboplatin, 660 patients received cisplatin, and 40 patients were exposed to both platinum agents. Most of these patients were male (2583 men [97.4%]), which is consistent with the overall VA population. Fifty percent of patients (1327 patients) were aged 60 to 69 years, followed by 35.5% (941 patients) aged 70 years and older. With regard to race, 2187 patients (82.5%) were White, and 239 (9.0%) were African American. Although 42.2% of patients with ES-SCLC (1120 patients) had unknown Eastern Cooperative Oncology Group (ECOG) performance status, most patients had performance status of 1 (709 patients [26.7%]), 2 (373 patients [14.1%]), and 0 (285 patients [10.7%]). Baseline characteristic of patients with ES-SCLC are described in eTable 1 in the Supplement.

**Figure 1.**
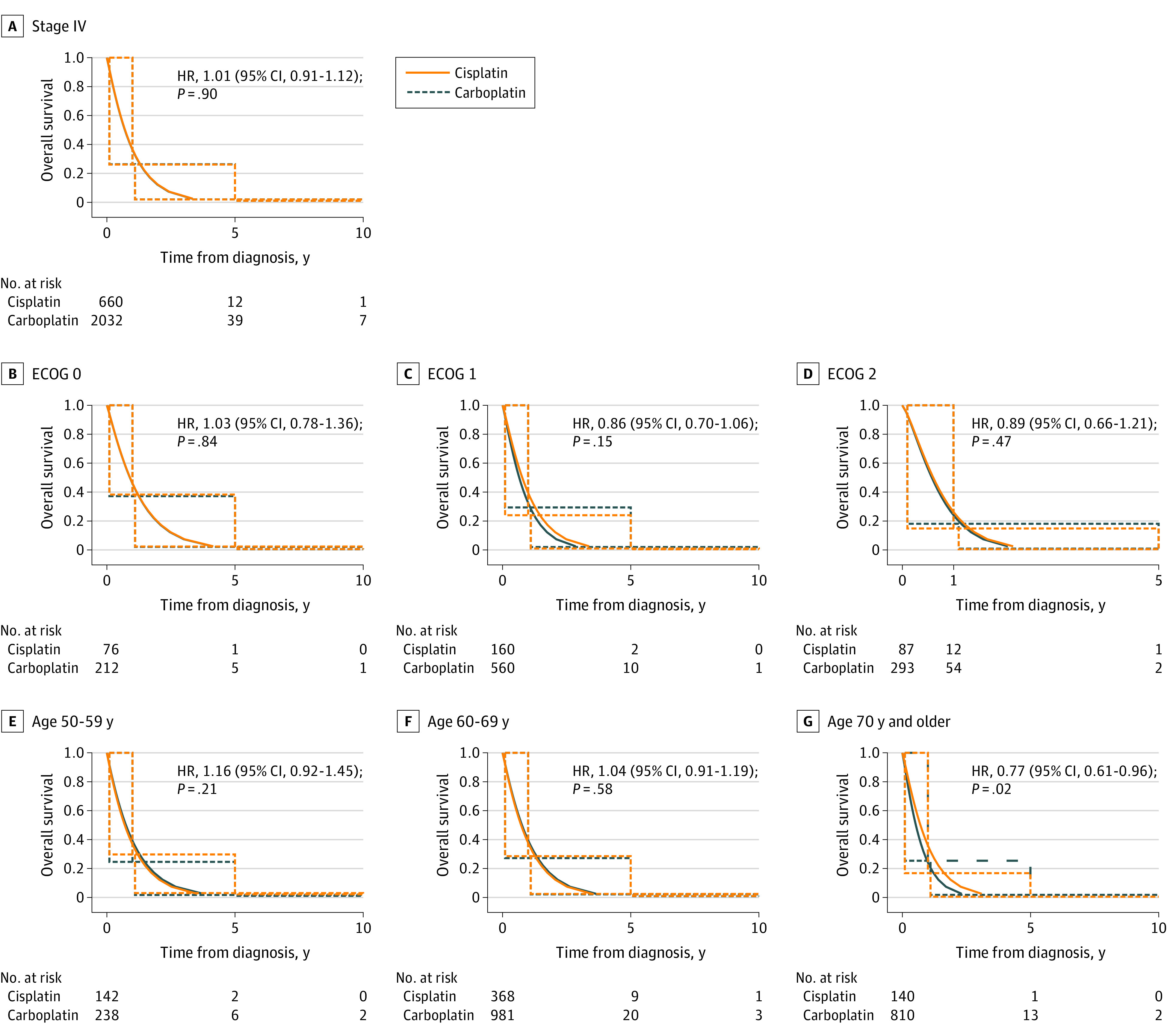
Overall Survival for Patients With Extended-Stage Small Cell Lung Cancer Receiving Cisplatin and Carboplatin by Cancer Stage, Eastern Cooperative Oncology Group (ECOG) Performance Status, and Age Graphs show overall survival for patients with cancer stage IV (A), by ECOG performance status (B-D), and by age group (E-G). The solid lines were estimated by a parametric Weibull proportional hazards regression analysis for interval censored data, and the dotted lines were generated by an interval-censored Cox proportional hazard model. HR indicates hazard ratio.

Among patients with ES-SCLC, the median OS was 8.45 months (95% CI, 7.75-9.20 months) for those treated with cisplatin and 8.51 months (95% CI, 8.07-8.97 months) for those treated with carboplatin. The corresponding HR between the 2 groups was not significant (HR, 1.01; 95% CI, 0.91-1.12; *P* = .90) (Figure 1A). Subset analysis was performed to identify groups of patients who might benefit from either regimen. When comparing different age groups, there was a survival difference in the group aged 70 years and older favoring carboplatin, as the median OS was 6.36 months (95% CI, 5.31-7.56 months) for cisplatin and 8.47 months (95% CI, 7.79-9.19 months) for carboplatin (HR, 0.77; 95% CI, 0.61-0.96; *P* = .02). There was no survival difference between the 2 agents in other age groups (Figure 1C). Furthermore, subgroup analysis showed that cisplatin and carboplatin led to similar median OS regardless of performance status (ECOG 0: HR, 1.03; 95% CI, 0.78-1.36; *P* = .84; ECOG 1: HR, 0.86; 95% CI, 0.70-1.06; *P* = .15; ECOG 2: HR, 0.89; 95% CI, 0.66-1.21; *P* = .47) Figure 1B). Table 1 displays the multivariable analysis of performance status and age, which showed no significant difference in survival between the 2 treatment groups with ES-SCLC (HR, 0.96; 95% CI, 0.83-1.10; *P* = .54). ECOG performance status in eTable 2 in the Supplement was an independent factor associated with survival regardless of which platinum agent was used, whereas age was not.

**Table 1.** Multivariable Cox Proportional Hazard Interval-Censored Regression Analysis of Factors Associated with Overall Survival in Patients With Extensive-Stage Small Cell Lung Cancer

Variable	Events, No./patients, No.	HR (95% CI)	*P* value
Age, y			
40-59	227/231	1 [Reference]	.67
≥ 60	1292/1327	0.97 (0.83-1.12)	
Eastern Cooperative Oncology Group performance status			
0	281/288	1 [Reference]	<.001
1-5	1238/1270	1.35 (1.18-1.54)	
Treatment			
Cisplatin	1 [Reference]	1 [Reference]	.54
Carboplatin	1171/1204	0.96 (0.83-1.10)	

Abbreviation: HR, hazard ratio.

### Patients With LS-SCLC

Among the 1756 patients with LS-SCLC (TNM stages I-III), 801 patients received carboplatin, 1018 individuals were treated with cisplatin, and 63 patients were exposed to both platinum agents. As expected in a military setting, most patients were male (1669 men [95.0%]). The most common age group was 60 to 69 years (935 patients [53.2%]) followed by patients aged 70 years and older (535 patients [30.5%]). Although 79.8% of the described population was White (1402 patients), African Americans constituted 12.1% of patients (212 patients). Most patients’ ECOG performance status was 1 (559 patients [31.8%]) and 0 (344 patients [19.6%]). The baseline characteristics of patients with LS-SCLC are summarized in eTable 2 in the Supplement.

**Figure 2.**
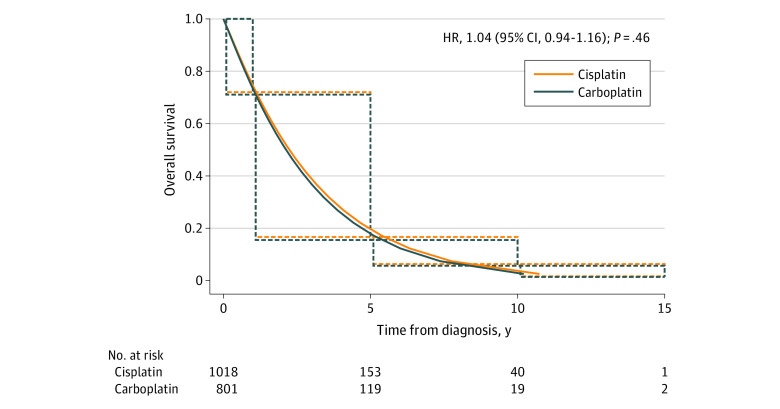
Overall Survival for Patients With Limited-Stage Small Cell Lung Cancer Receiving Cisplatin and Carboplatin The solid lines were estimated by a parametric Weibull proportional hazards regression analysis for interval censored data, and the dotted lines were generated by an interval-censored Cox proportional hazard model. HR indicates hazard ratio.

**Figure 3.**
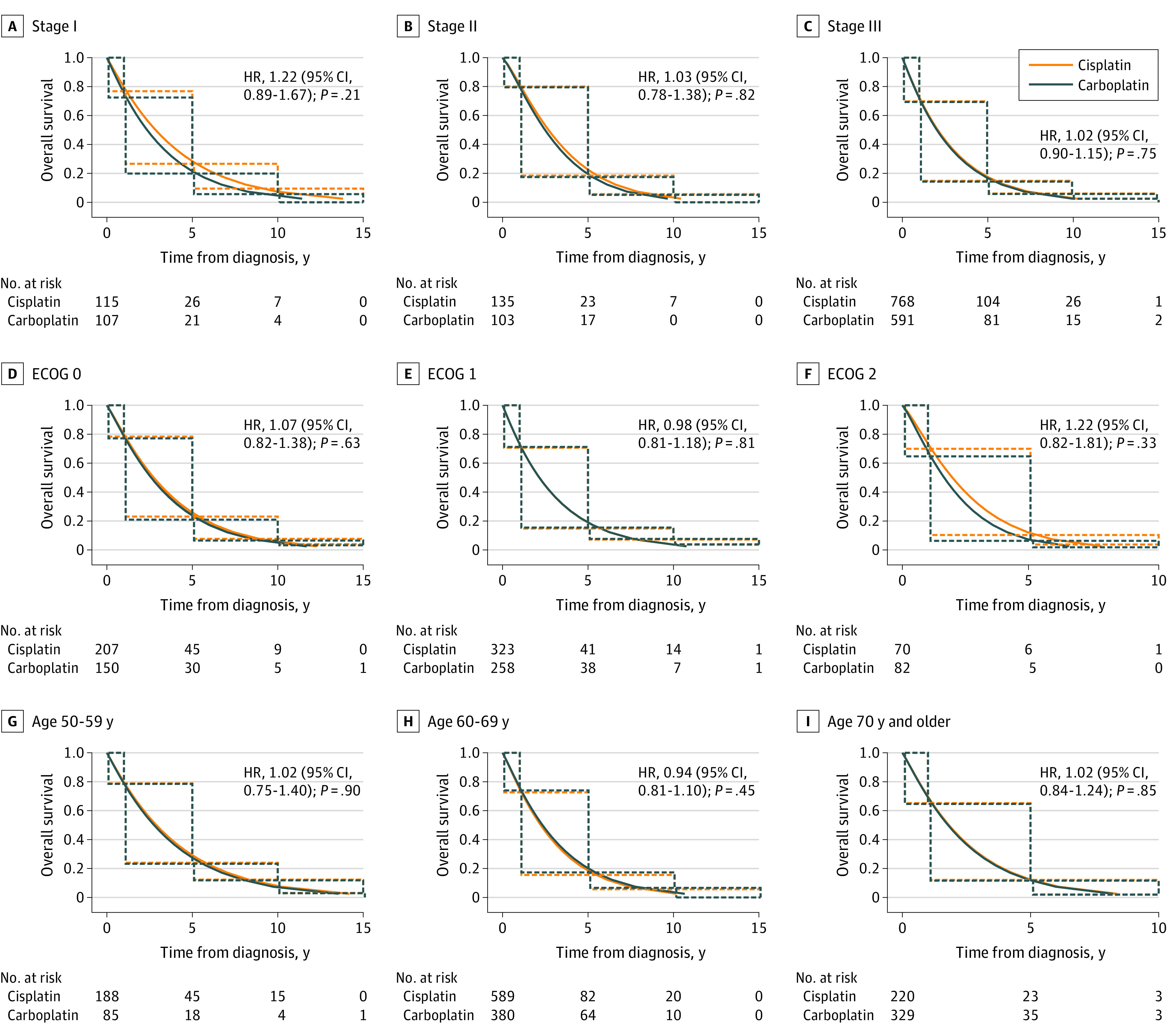
Overall Survival for Patients With Limited-Stage Small Cell Lung Cancer Receiving Cisplatin and Carboplatin by Cancer Stage, Eastern Cooperative Oncology Group (ECOG) Performance Status, and Age Graphs show overall survival by cancer stage (A-C), ECOG performance status (D-F), and age (G-I). The solid lines were estimated by a parametric Weibull proportional hazards regression analysis for interval censored data, and the dotted lines were generated by an interval-censored Cox proportional hazard model. HR indicates hazard ratio.

Figure 2 demonstrates the median OS of patients with LS-SCLC treated with carboplatin-based regimen compared with cisplatin. There was no significant difference between the OS with cisplatin (median, 26.92 months; 95% CI, 25.03-28.81 months) compared with carboplatin (median, 25.58 months; 95% CI, 23.64-27.72 months) (HR, 1.04; 95% CI, 0.94-1.16; *P* = .46). Moreover, the median OS was not different between the 2 agents on the basis of stage (I-III), ECOG performance status, and age group (Figure 3). A multivariable Cox proportional hazard interval-censored regression analysis of factors related to OS accounting for stage (I-III), ECOG performance status, and age did not demonstrate a significant difference in survival between carboplatin-based and cisplatin-based chemotherapy in patients with LS-SCLC (HR, 0.995; 95% CI, 0.86-1.15; *P* = .95) (Table 2). Younger age and better performance status were associated with longer survival regardless of the platinum agent used.

**Table 2.** Multivariable Cox Proportional Hazard Interval-Censored Regression Analysis of Factors Associated With Overall Survival Limited-Stage Small Cell Lung Cancer

Variable	Events, No./patients, No.	HR (95% CI)	*P* value
Age, y			
30-59	146/188	1 [Reference]	<.001
≥ 60	802/944	1.55 (1.28-1.88)	
Eastern Cooperative Oncology Group performance status			
0	297/357	1 [Reference]	.004
1-4	651/775	1.24 (1.07-1.44)	
Stage			
I	109/135	1 [Reference]	NA
II	137/162	0.99 (0.77-1.26)	.91
III	702/835	1.14 (0.93-1.40)	.20
Treatment			
Cisplatin	513/613	1 [Reference]	.95
Carboplatin	435/519	0.995 (0.86-1.15)	

Abbreviation: HR, hazard ratio.

## Discussion

The choice of carboplatin or cisplatin in the chemotherapy doublet backbone for the treatment of SCLC is an ongoing debate in current clinical practice. This retrospective cohort study found that cisplatin-based chemotherapy was not associated with a survival advantage over carboplatin-based therapy for patients with either ES-SCLC or, interestingly, LS-SCLC. Consistent with its more toxic profile, cisplatin-based palliative chemotherapy was associated with worse survival in the subset of ES-SCLC patients older than 70 years. All other subgroup analyses showed no survival differences between the 2 chemotherapy regimens including in young and fit patients. Multivariable analysis accounting for age, stage, and performance status similarly showed no difference between both platinum agents. Performance status was associated with better OS in both LS-SCLC and ES-SCLC, regardless of the platinum agent used.

Traditionally, cisplatin-based treatment has been the standard of care for LS-SCLC, with the goal of cure unless cisplatin is contraindicated or the patient develops intolerable adverse effects.^5^ This recommendation by NCCN and other organizations is based on its perceived superiority to carboplatin even though it is associated with increased renal, neurologic, and emetogenic toxic effects and requires prolonged intravenous hydration during administration.^10^ Cisplatin is also favored in similarly aggressive solid tumors especially in the curative setting. For example, the standard of care treatment for muscle-invasive bladder cancer centers around cisplatin-based neoadjuvant chemotherapy.^19,20^ NCCN prefers cisplatin-based adjuvant therapy for stage II and III resected non–small cell lung cancer.^21,22,23^ In addition, in scenarios where organ preservation is sought, cisplatin is the preferred agent in concurrent chemotherapy in both head and neck and bladder cancers.^24,25,26^ The rationale of using platinum agents in SCLC is based on their antitumor activity resulting from DNA damage. These platinum-induced DNA adducts are recognized as DNA damage. The inability of the cancer cells to repair this damage using its multiple pathways (eg, nucleotide excision repair, base excision repair, and nonhomologous end joining) ultimately leads to apoptosis.^27^ SCLC is enriched in mutations in DNA-damage repair genes, including loss of function in *RAD51D*, *CHEK1*, and *BRCA2*.^28^ Accumulating data in several tumors (ovarian, breast, prostate, and pancreatic cancers) have shown that inherited or somatic mutations in DNA repair genes are particularly sensitive to platinum-based chemotherapy.^29^ For patients with LS-SCLC, thoracic radiation therapy improves survival, and platinum agents have shown radiotherapy potentiation effects.^30,31^ Given the similar effect of cisplatin and carboplatin on DNA damage repair system and the low survival rate of patients with LS-SCLC (5-year OS rate of approximately 20%), it is not surprising to find no difference in survival with carboplatin and cisplatin. In addition, cisplatin carries a boxed warning in the US for dose reduction for nephrotoxicity. The use of the less toxic carboplatin might allow for a more timely and complete administration of concurrent chemoradiation.

Current literature demonstrates a lack of superiority of cisplatin vs a carboplatin-based regimen in ES-SCLC. The COCIS meta-analysis^15^ of individual patient data showed that carboplatin-based regimens appear to be equally effective in terms of OS, progression-free survival, and overall response rate compared with cisplatin-based combinations for the first-line therapy of SCLC, differing only in their toxicity profiles. However, the 4 randomized trials^11,32,33,34^ included were limited by small sample size and heterogeneity of chemotherapy regimens, and carboplatin is still used frequently in some settings (approximately 25% in the current study). To our knowledge, this study is the largest study to date to add to the body of literature supporting the use of carboplatin in ES-SCLC, particularly in young and fit patients.

In a phase 2 trial,^12^ carboplatin demonstrated clinical efficacy in LS-SCLC when combined with paclitaxel or etoposide that was similar to historical data with cisplatin-based regimens. Survival rates similar to those with the cisplatin-based regimen were identified in a Minnie Pearl Cancer Research Network study.^13^ Despite these small phase 2 trials, the current preferred NCCN recommendation in LS-SCLC is cisplatin as the radiosensitizer of choice. To our knowledge, this study is the first to compare carboplatin and cisplatin in LS-SCLC and shows that the more popular cisplatin carries no survival advantage. Large-scale prospective studies are needed to definitely compare carboplatin-based and cisplatin-based regimens for the treatment of SCLC. However, the favorable toxicity profile of carboplatin and comparable OS support its use in both LS-SCLC and ES-SCLC. This should inform both clinical practice and clinical trial design in SCLC as immune checkpoint inhibitors are evaluated in LS-SCLC and new agents are tested in combination with a platinum doublet in ES-SCLC.^35^

### Limitations

This study is limited by its retrospective nature, which carries risk of bias. The studied VA population might also not be representative of the population at large, as it almost exclusively consists of male and older patients. We acknowledge the lack of availability of data on radiation dosing, chemotherapy dosing and interruptions, and lack of follow-up and adverse effects. Data input on survival into the registry was not optimal. Forty patients with ES-SCLS and 63 patients with LS-SCLC in our database received both carboplatin and cisplatin, which carries the risk of confounding by immortal time bias. In retrospective observational interval-censored data, it is not possible to eliminate confounding by indication and/or residual confounding. Although we did adjust for confounding by indication using a conventional multivariable analysis, our method may not have completely ruled out confounding. As our study did not have access to individual patient records, we are unable to ascertain the reason behind the switch from one platinum agent to the other happened. Because of limitations in database construction, we were unable to exclude them from our analysis.

## Conclusions

 This cohort study found that cisplatin was not associated with a survival advantage over carboplatin in patients with either ES-SCLC or LS-SCLC, but carboplatin has a favorable toxicity profile. The favorable toxicity profile of carboplatin may allow more room for combination with novel treatment strategies.

## Notes

Supplement. **eTable 1. **Baseline Characteristics of ES-SCLC **eTable 2.** Baseline Characteristics of LS-SCLC
