# Errors in Figure and Tables

**DOI:** 10.1001/jamanetworkopen.2022.46257

**Published:** 2023-01-20

In the Original Investigation titled “Comparison of Carboplatin With Cisplatin in Small Cell Lung Cancer in US Veterans,”^1^ published October 20, 2022, there were errors in the tables and a figure. The number of events and total patients receiving cisplatin treatment in Table 1 should have appeared as 348/354, and “NA” was incorrectly included in the column of *P* values in Table 2. In Figure 1, the colors of the lines in Panels B through G were inadvertently transposed. This article has been corrected.^1^
