# Supplementary material for: Comparison of Carboplatin With Cisplatin in Small Cell Lung Cancer in US Veterans
Source: JAMA Netw Open. 2022 Oct 20;5(10):e2237699. doi: 10.1001/jamanetworkopen.2022.37699 (PMC9585434; doi:10.1001/jamanetworkopen.2022.37699)
Supplement: Supplement. — eTable 1. Baseline Characteristics of ES-SCLC eTable 2. Baseline Characteristics of LS-SCLC [file jamanetwopen-e2237699-s001.pdf]

Supplemental Online Content

Azar I, Yazdanpanah O, Jang H, et al. Comparison of carboplatin with cisplatin in small cell lung cancer in US veterans. *JAMA Netw Open.* 2022;5(10):e2237699. doi:10.1001/jamanetworkopen.2022.37699

eTable 1. Baseline Characteristics of ES-SCLC
eTable 2. Baseline Characteristics of LS-SCLC

This supplemental material has been provided by the authors to give readers additional information about their work.

© 2022 Azar I et al. *JAMA Network Open.*

eTable 1. Baseline Characteristics of ES-SCLC

	All (n=2652)*	Carboplatin (n=2032)	Cisplatin (n=660)	P value[#]
**Gender – no. (%)**				0.008
Female	69 (2.6%)	45 (2.2%)	28 (4.2%)	
Male	2583 (97.4%)	1987 (97.8%)	632 (95.8%)	
**Age – no. (%)**				<0.001
40-49 years	13 (0.5%)	3 (0.1%)	10 (1.5%)	
50-59 years	371 (14.0%)	238 (11.7%)	142 (21.5%)	
60-69 years	1327 (50.0%)	981 (48.3%)	368 (55.8%)	
≥70 years	941 (35.5%)	810 (39.9%)	140 (21.2%)	
**Race – no. (%)**				
African American	239 (9.0%)	183 (9.0%)	62 (9.4%)	0.82
White	2187 (82.5%)	1684 (82.9%)	534 (80.9%)	
Other[¥]	49 (1.8%)	38 (1.9%)	11 (1.7%)	
Declined to Answer/Unknown	177 (6.7%)	127 (6.2%)	53 (8%)	
**Ethnicity – no. (%)**				0.22
Hispanic or Latino	39 (1.5%)	26 (1.3%)	13 (2.0%)	
Not Hispanic or Latino	2489 (93.9%)	1919 (94.4%)	609 (92.3%)	

© 2022 Azar I et al. *JAMA Network Open.*

Declined to Answer/Unknown	124 (4.7%)	87 (4.3%)	38 (5.8%)	
**Performance status – no. (%)**				0.46
ECOG 0	285 (10.7%)	212 (10.4%)	76 (11.5%)	
ECOG 1	709 (26.7%)	560 (27.6%)	160 (24.2%)	
ECOG 2	373 (14.1%)	293 (14.4%)	87 (13.2%)	
ECOG 3	150 (5.7%)	127 (6.2%)	28 (4.2%)	
ECOG 4	11 (0.4%)	9 (0.4%)	2 (0.3%)	
ECOG 5	4 (0.2%)	3 (0.1%)	1 (0.2%)	
Unknown	1120 (42.2%)	828 (40.7%)	306 (46.4%)	

[*] 40 patients received both carboplatin and cisplatin;

[#] Fisher's exact test

[¥] Asian, American Indian or Alaskan Native, Native Hawaiian or other Pacific Islander, and multiple race

© 2022 Azar I et al. *JAMA Network Open.*

eTable 2. Baseline Characteristics of LS-SCLC

	All (n=1756)*	Carboplatin (n=801)	Cisplatin (n=1018)	P value[#]
**Gender – no. (%)**				0.83
Female	87 (5.0%)	39 (4.9%)	52 (5.1%)	
Male	1669 (95.0%)	762 (95.1%)	966 (94.9%)	
**Age – no. (%)**				<0.001
30-39 years	2 (0.1%)	0 (0%)	2 (0.2%)	
40-49 years	24 (1.4%)	7 (0.9%)	19 (1.9%)	
50-59 years	260 (14.8%)	85 (10.6%)	188 (18.5%)	
60-69 years	935 (53.2%)	380 (47.4%)	589 (57.9%)	
≥70 years	535 (30.5%)	329 (41.1%)	220 (21.6%)	
**Race – no. (%)**				0.42
African American	212 (12.1%)	95 (11.9%)	125 (12.3%)	
White	1402 (79.8%)	651 (81.3%)	800 (78.6%)	
Other[¥]	28 (1.6%)	10 (1.2%)	18 (1.8%)	
Declined to Answer/Unknown	114 (6.5%)	45 (5.6%)	75 (7.4%)	
**Ethnicity – no. (%)**				0.16
Hispanic or Latino	30 (1.7%)	8 (1.0%)	22 (2.2%)	
Not Hispanic or Latino	1653 (94.1%)	759 (94.8%)	953 (93.6%)	
Declined to Answer/Unknown	73 (4.2%)	34 (4.2%)	43 (4.2%)	

© 2022 Azar I et al. *JAMA Network Open.*

Performance status – no. (%)				0.002
ECOG 0	344 (19.6%)	150 (18.7%)	207 (20.3%)	
ECOG 1	559 (31.8%)	258 (32.2%)	323 (31.7%)	
ECOG 2	146 (8.3%)	82 (10.2%)	70 (6.9%)	
ECOG 3	38 (2.2%)	27 (3.4%)	12 (1.2%)	
ECOG 4	3 (0.2%)	2 (0.2%)	1 (0.1%)	
Unknown	666 (37.9%)	282 (35.2%)	405 (39.8%)	

[*] 63 patients received both carboplatin and cisplatin

[#] Fisher's exact test

[¥]Asian, American Indian or Alaskan Native, and Native Hawaiian or other Pacific Islander

© 2022 Azar I et al. *JAMA Network Open.*
